# An integrative approach to identify sand fly vectors of leishmaniasis in Ethiopia by morphological and molecular techniques

**DOI:** 10.1186/s13071-020-04450-2

**Published:** 2020-11-17

**Authors:** Myrthe Pareyn, Vit Dvorak, Petr Halada, Natalie Van Houtte, Nigatu Girma, Wim de Kesel, Behailu Merdekios, Fekadu Massebo, Herwig Leirs, Petr Volf

**Affiliations:** 1 Evolutionary Ecology Group, Biology Department, University of Antwerp, Antwerp, Belgium; 2 Department of Parasitology, Faculty of Science, Charles University, Prague, Czech Republic; 3 BioCeV-Institute of Microbiology, The Czech Academy of Sciences, Vestec, Czech Republic; 4 Biology Department, Arba Minch University, Arba Minch, Ethiopia; 5 Department of Public Health, Arba Minch University, Arba Minch, Ethiopia

**Keywords:** Sand flies, *Phlebotomus*, Morphology, DNA barcoding, MALDI-TOF mass spectrometry, Protein profiling, Ethiopia

## Abstract

**Background:**

Ethiopia is affected by human leishmaniasis caused by several *Leishmania* species and transmitted by a variety of sand fly vectors of the genus *Phlebotomus*. The sand fly fauna in Ethiopia is highly diverse and some species are closely related and similar in morphology, resulting in difficulties with species identification that requires deployment of molecular techniques. DNA barcoding entails high costs, requires time and lacks reference sequences for many Ethiopian species. Yet, proper species identification is pivotal for epidemiological surveillance as species differ in their actual involvement in transmission cycles. Recently, protein profiling using MALDI-TOF mass spectrometry has been introduced as a promising technique for sand fly identification.

**Methods:**

In our study, we used an integrative taxonomic approach to identify most of the important sand fly vectors of leishmaniasis in Ethiopia, applying three complementary methods: morphological assessment, sequencing analysis of two genetic markers, and MALDI-TOF MS protein profiling.

**Results:**

Although morphological assessment resulted in some inconclusive identifications, both DNA- and protein-based techniques performed well, providing a similar hierarchical clustering pattern for the analyzed species. Both methods generated species-specific sequences or protein patterns for all species except for *Phlebotomus pedifer* and *P. longipes*, the two presumed vectors of *Leishmania aethiopica*, suggesting that they may represent a single species, *P. longipes* Parrot & Martin. All three approaches also revealed that the collected specimens of *Adlerius* sp. differ from *P.* (*Adlerius*) *arabicus*, the only species of *Adlerius* currently reported in Ethiopia, and molecular comparisons indicate that it may represent a yet undescribed new species.

**Conclusions:**

Our study uses three complementary taxonomical methods for species identification of taxonomically challenging and yet medically import Ethiopian sand flies. The generated MALDI-TOF MS protein profiles resulted in unambiguous identifications, hence showing suitability of this technique for sand fly species identification. Furthermore, our results contribute to the still inadequate knowledge of the sand fly fauna of Ethiopia, a country severely burdened with human leishmaniasis.
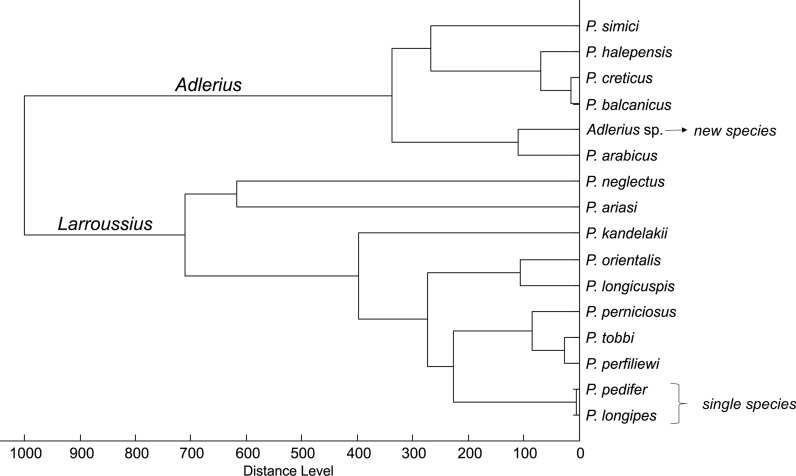

## Background

Phlebotomine sand flies (Diptera, Phlebotominae) are hematophagous insects of great medical importance as the females of some species are the vectors of the protozoans *Leishmania* spp., which are transmitted during blood-feeding on a vertebrate host. Human leishmaniasis manifests in three major clinical forms: visceral (VL), cutaneous (CL), and mucocutaneous (MCL) leishmaniasis [1, 2].

All three forms affect people in Ethiopia, particularly the poorest part of the population living in rural areas [3, 4]. VL is generally endemic in the lowlands widespread in the country and is caused by *Leishmania donovani*. Proven vectors of VL in different regions in Ethiopia are *Phlebotomus orientalis*, *P. martini* and to a lesser extent *P. celiae* [5–9]. Localized and diffuse forms of CL, and MCL occur mainly at mid-highland altitudes, on the mountain ridges of the Ethiopian Rift Valley. The main causative parasite species is *L. aethiopica*, which is transmitted by *P. longipes* in northern and central Ethiopia and *P. pedifer* in southwestern Ethiopia [10–16].

It is pivotal for entomological and eco-epidemiological research to accurately identify the sand fly species that act as vectors in a particular area and assess their ecology and behavior, as this information is a prerequisite for implementation of efficient, targeted control programmes [17].

Morphological identification is performed by examination of the mounted head and abdomen of the sand flies which bear the main distinctive characteristics for classification (cibarial and pharengeal armature, genitalia). However, this taxonomic approach is labor-intensive, demands high proficiency, and morphological keys are often obsolete and incomprehensive, leading to incorrect identifications [18–20].

To overcome these drawbacks, studies are frequently shifting to molecular techniques, mostly DNA sequencing analyses which allow simultaneous processing of samples, provide reliable identifications and have good reference sequences available for many species. Yet, this approach is technically demanding, considered costly for large-scale studies and its validity depends on the genetic variability of the target locus [21, 22].

Recently, an alternative molecular method, protein profiling by matrix-assisted laser desorption/ionization time-of-flight mass spectrometry (MALDI-TOF MS), has been introduced for species identification in different arthropod families, including sand flies [20, 23–28]. This approach provides unique protein profiles that allow unambiguous species identification and sample processing is rapid, simple and cost-effective. However, it still needs validation on field specimens and requires a centralized database to approve its use for routine species identification of sand flies [20, 29]. As the method utilizes only certain body parts, in sand flies particularly the thorax in standardized protocols [20], a coordinated sample preparation allows simultaneous or later application of other techniques, including morphological analysis of the mounted head and genitalia and DNA-based techniques using template DNA isolated from the sand fly abdomen.

The sand fly fauna in Ethiopia is very diverse, comprising remarkable numbers of species in both genera *Sergentomyia* and *Phlebotomus*, within which species of at least six subgenera were recorded, many of them proven or suspected vectors of several *Leishmania* species and thus of great medical significance. Among these, some closely related sand fly species are remarkably similar in morphological characteristics, resulting in many difficulties for conclusive species identification [30–33]. Especially females of some species are challenging to distinguish from each other as species-specific morphological features are poorly described or undefined. These species, however, often do not play the same role in *Leishmania* transmission which is hindering sand fly ecology studies and implementation of control measures accordingly, indicating that more sophisticated techniques are necessary for species differentiation [30, 31]. DNA barcoding could be an appropriate alternative, although very few reference sequences of the Ethiopian sand fly species are currently available in genetic databases and analyses are rather too costly to process large sample sizes.

Our study presents for the first time an integrative taxonomic approach to identify most of the important sand fly vectors of leishmaniasis in Ethiopia, applying three complementary methods: morphological assessment, sequencing analysis of two genetic markers, and MALDI-TOF MS protein profiling. We aimed to demonstrate that MALDI-TOF MS protein profiling could be a suitable taxonomical technique, providing unambiguous sand fly species identification in Ethiopia. Furthermore, our results contribute to the currently still inadequate knowledge of the sand fly fauna in Ethiopia, a region among those most affected by the burden of human leishmaniasis.

## Methods

### Sand flies

**Fig. 1:**
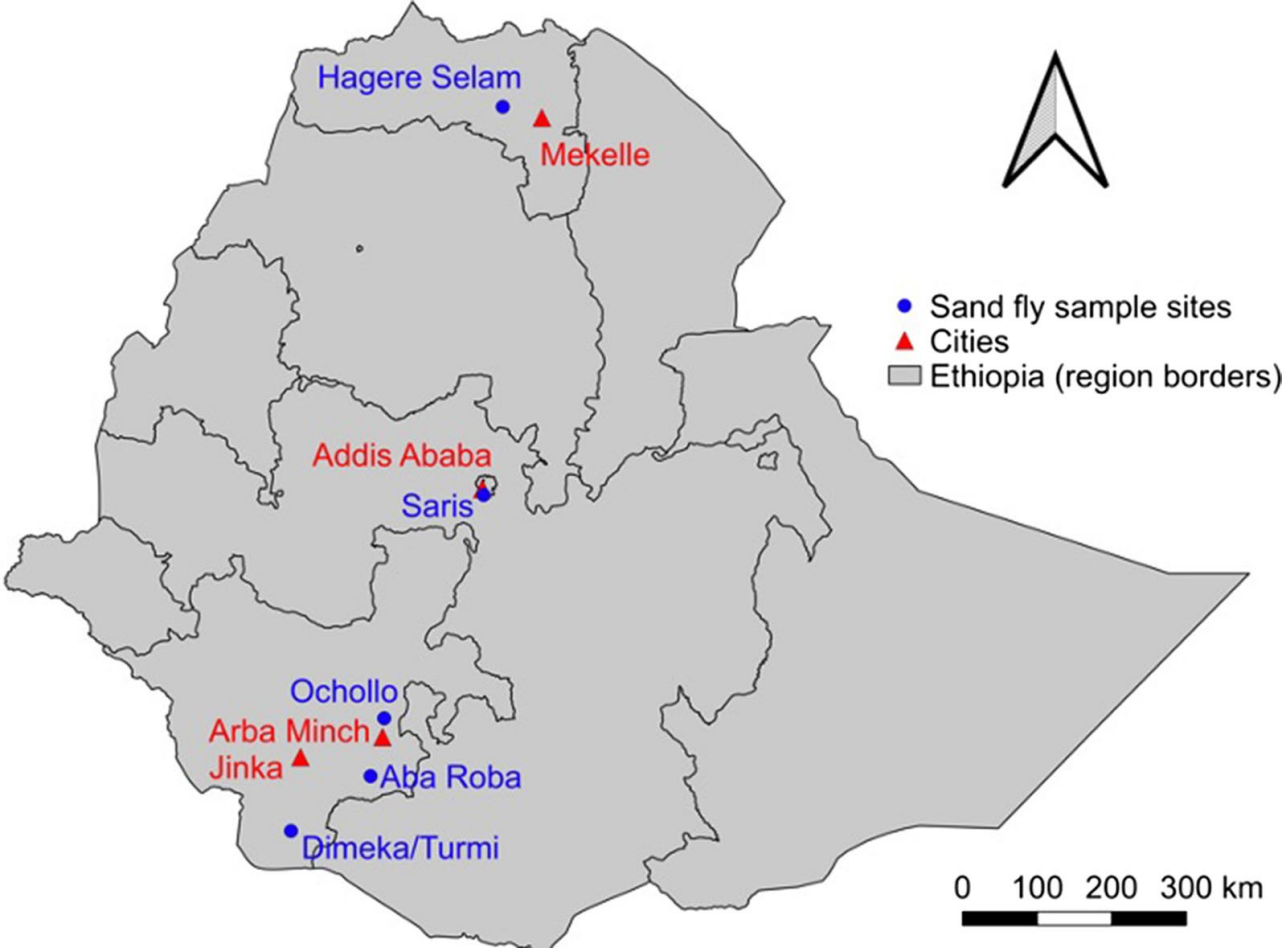
Sampling locations of field collected sand flies [66, 67]. Blue dots represent the places where field collected sand flies came from. CL foci: Hagere Selam (Tigray Region); Saris (Addis Ababa city administration); and Ochollo (Southern Nations, Nationalities and Peoples’ Region, SNNPR). VL foci: Aba Roba, Dimeka and Turmi (SNNPR). The cities nearby the sample sites are displayed by red triangles

Field specimens were captured in May and September 2019 from different CL (Hagere Selam in the north, Saris in the center and Ochollo in the southwest) and VL (Aba Roba, Dimeka and Turmi in the south) foci in Ethiopia (Fig. 1). Sand flies were captured with CDC miniature light traps (John W. Hock Company, Gainesville, Florida, USA), which were set at 18:00 h and collected at 7:00 h the next morning. In order to capture CL vectors in the mid-highlands, traps were placed in caves or around rocky areas, whereas to capture VL vectors in the lowlands, traps were set nearby termite hills and human dwellings [11, 34, 35]. Collected sand flies were preserved in 70% ethanol and stored at −20 °C [36].

### Morphological identification of sand fly species

The head and terminal segments of the abdomen of the sand fly specimens were mounted using CMCP-10 high viscosity mounting medium (Polysciences, Hirschberg, Germany) and species identification was done according to relevant morphological keys [30–32, 37–42]. Slide-mounted specimens were observed using a light microscope Olympus BX51 (Olympus Life Science, Waltham, USA) with a camera system Olympus D70. Morphological characters were measured using the QuickPHOTO MICRO 3.0 software (Promicra, Prague, Czech Republic).

### Molecular identification of sand fly species

After mounting, the remaining part of the abdomen was used for molecular identification of the sand fly species. DNA was isolated using the QIAamp DNA Mini Kit (Qiagen, Hilden, Germany). Samples were subsequently subjected to two PCRs targeting (i) a fragment of the cytochrome *c* oxidase subunit 1 (*cox*1) with primers LCO 1490 (5′-GGT CAA ATC ATA AAG ATA TTG G-3′) and HCO 2198 (5′-TAA ACT TCA GGG TGA CCA AAA AAT CA-3′) in a total reaction volume of 25 µl as described by Folmer et al. [43]; and (ii) the *nad*4 gene with primers ND4ar (5′-AAR GCT CAT GTT GAA GC-3′) and ND4c (5′-ATT TAA AGG YAA TCA ATG TAA-3′) in a reaction volume of 50 µl based on Soto et al. [44]. Amplicons were visualized on a 1% agarose gel and obtained products were purified with the QIAquick PCR Purification Kit (Qiagen). These were sent to Vlaams Instituut voor Biotechnologie (VIB; University of Antwerp, Wilrijk, Belgium) or BIOCEV OMICS genetika (Charles University, Vestec, Czech Republic) for Sanger sequencing in two directions with the primers used for DNA amplification.

### Phylogenetic analyses

The obtained chromatograms were edited and consensus sequences were generated for each specimen, which were compared with reference sequences in GenBank using BLAST. Multiple sequence alignment of the *cox*1 and *nad*4 sequences was performed using the Clustal W tool implemented in MEGA X 10.1 and primers were trimmed to have sequences with an equal length of 658 bp and 597 bp, respectively [45, 46]. The nucleotide composition and sequence divergence were calculated with the Kimura 2-parameter model (K2P) and a distance matrix was generated [47, 48]. A neighbor-joining (NJ) tree of the K2P distances was created using a bootstrapping method with 1000 replicates for a graphical presentation of the clustering pattern of the sand fly species [46, 49]. To generate a NJ tree for our specimens of the subgenus *Adlerius* in combination with other species of the same subgenus, *cox*1 sequences were retrieved from GenBank. Based on the NJ trees, groups of species were indicated to assess the inter- and intra-species distances.

### MALDI-TOF MS analysis of sand fly species

The MALDI matrix was prepared fresh as an aqueous 60% acetonitrile/0.3% TFA solution of sinapinic acid (30 mg/ml; Bruker Daltonics, Bremen, Germany). Specimens stored in 70% ethanol were air-dried, dissected and thoraces were manually ground in 10 μl of 25% formic acid by a BioVortexer homogenizer (BioSpec, Bartlesville, USA) with sterile disposable pestles. After a short centrifugation of the homogenate (10,000×*g* for 15 s), 2 µl was mixed with 2 µl of MALDI matrix and 1 µl was deposited and air-dried on a steel target plate (Bruker Daltonics) in duplicate. Protein mass spectra were measured in a mass range of 4–25 kDa by an Autoflex Speed MALDI-TOF spectrometer (Bruker Daltonics) and calibrated externally with the Bruker Protein Calibration Standard I. Each spectrum was acquired as a sum of 4000 manually adjusted laser shots (20 × 200 shots from different positions of the target spot) and visualized by FlexAnalysis 3.4 software (Bruker Daltronics).

For cluster analysis, the protein profiles were processed (normalization, smoothing, baseline subtraction and peak picking) using MALDI Biotyper 3.1. (Bruker Daltronics). The peak picking parameters for generation of a main spectrum (MSP) were to include maximum 100 peaks, which had a signal-to-noise ratio greater than 3 and a relative intensity of minimum 1% of the most intense peak. The desired peak frequency for MSP reference spectra was set to 60%. For MSP dendrogram creation, a correlation distance measure and average linkage parameters were applied. The dendrograms were generated using the individual MSPs or MSP references created for each sand fly species. In addition to six field-caught Ethiopian species, MSP references from our sand fly database were included (country of origin is given in parentheses): *P. ariasi* (France); *P. kandelakii* (Georgia); *P. longicuspis* (Morocco); *P. neglectus* (Croatia); *P. orientalis* (Ethiopia); *P. perfiliewi* (Macedonia); *P. perniciosus* (Spain); *P. tobbi* (Northern Macedonia); *P. arabicus* (Israel); *P. balcanicus* (Georgia); *P. creticus* (Crete); *P. halepensis* (Georgia); *P. simici* (Macedonia). *Phlebotomus arabicus* and *P. orientalis* originated from colonies maintained in Prague, the others were field-collected.

## Results

### Morphological species identification

**Fig. 2:**
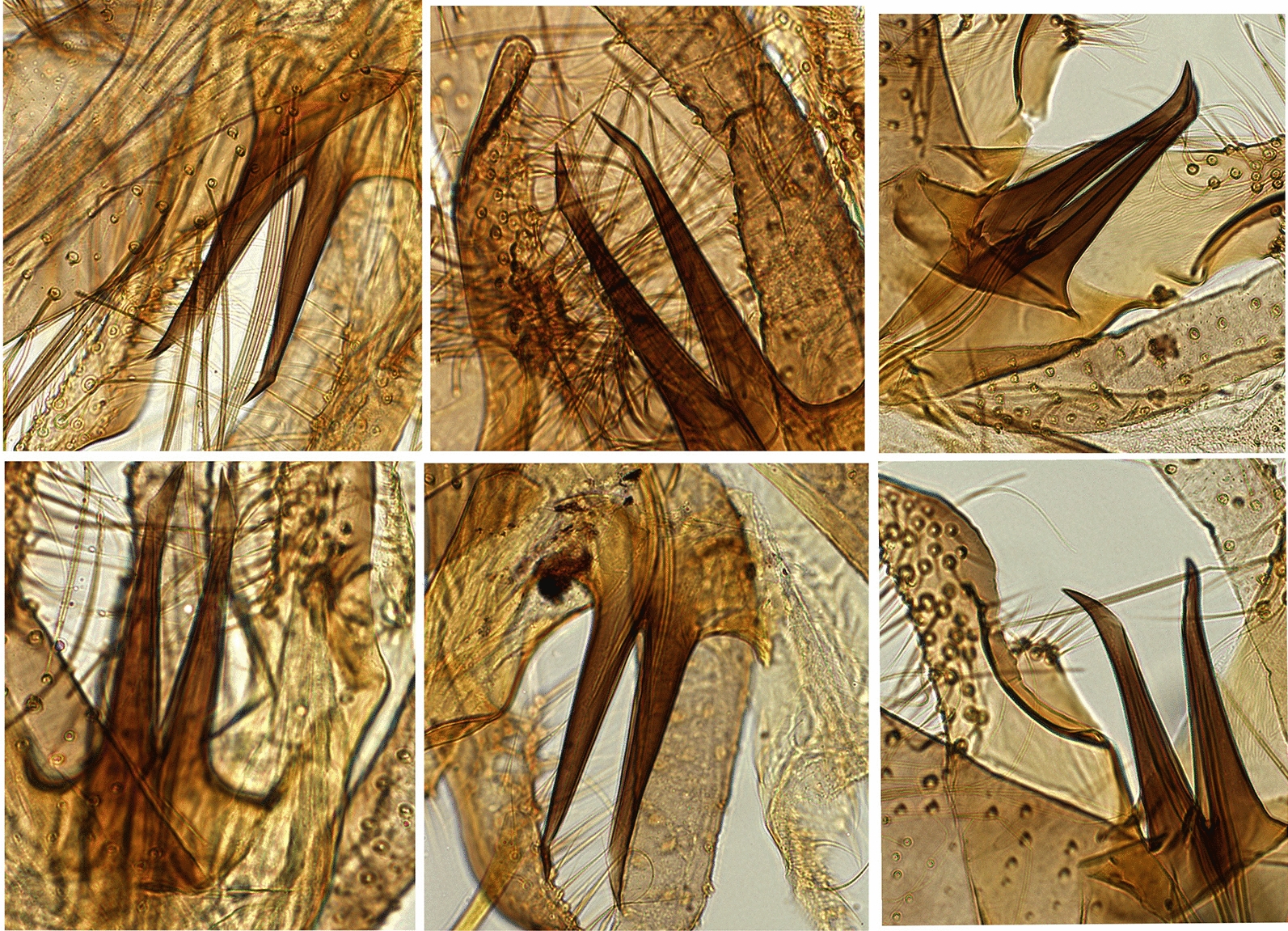
Aedeagi of male *Phlebotomus longipes* (left two columns 1 and 2) and *P. pedifer* (right column 3). *Phlebotomus longipes* was acquired for Hagere Selam and Saris (northern and central Ethiopia) and *P. pedifer* from Ochollo (southern Ethiopia)

Morphological analysis identified six sand fly species of four subgenera. Three species, *P.* (*Phlebotomus*) *duboscqi*, *P.* (*Synphlebotomus*) *celiae* and *P.* (*Sy.*) *martini*, were identified in VL endemic sites in the south (Dimeka, Turmi and Aba Roba). *Phlebotomus* (*Larroussius*) *pedifer* was collected in Ochollo and *P.* (*La.*) *longipes* in Saris and Hagere Selam. A main feature to differentiate the two species of the subgenus *Larroussius* is based on the bending of the tip of the male aedeagus [31, 41]. However, our specimens showed a varying range of tip endings within a single species (Fig. 2) that did not allow clear differentiation between the species-specific morphological arrangements of the two species. Furthermore, we counted overlapping numbers of inner surface coxite hairs, being 39 (31–48, *n* = 43) for *P. pedifer* from Ochollo, 31 (29–36, *n* = 13) for *P. longipes* from Hagere Selam and 44 (40–48, *n* = 2) for *P. longipes* from Saris. Therefore, species identification of the two *Larroussius* species was mainly based on previous geographical presence records [10, 11, 34].

Females of the species within the subgenera *Synphlebotomus* and *Larroussius* could often not be separated to species level based on morphological features [30, 41, 50, 51].

Seven males and one female of an additional species were captured from a single cave nearby Hagere Selam (13° 40′14″ N, 39° 07′ 29″ E) and identified as a species of the subgenus *Adlerius* (further referred to as ‘*Adlerius* sp.’). Morphometric analysis of four male specimens of *Adlerius* sp. (Table 1) indicates that our sand flies show high similarity in the number and position of coxite hairs and the aedeagus tip to tooth distance as described for *P. davidi* [37]. The style, coxite and aedeagus length of our specimens, however, are longer than reported for *P. davidi*, but shorter than measured and described for *P. arabicus* and fit more to *P. naqbenius*. Accordingly, no conclusive species identification could be reached for *Adlerius* sp.

**Table 1:** Morphometric analysis of the *Adlerius* sp. from Ethiopia, in comparison with other *Adlerius* species

Species (country, reference)	Ascoid formula	Style length	Coxite length	No. of coxite hairs	Position coxite hairs	Aedeagus length	Aedeagus tip to tooth
*Adlerius* sp.^a^ (Ethiopia, Pareyn)	2/3-7, 1/8-15	173 (158–188)	366 (355–387)	45 (36–52)	0.56 (0.51–0.57)	186 (181–195)	13 (11–15)
*P. davidi* (Yemen & Ethiopia, Artemiev [37])	2/3-7, 1/8-15	157 (156–160)	315 (300–328)	46 (38–59)	0.59 (0.55–0.64)	162 (152–172)	12 (10–14)
*P. arabicus* (Israel, PV, unpublished)	2/3-7	189 (176–206)	386 (370–428)	64 (55–76)	–	183 (176–206)	20 (17–25)
*P. arabicus* TYPE^b^ (Saudi Arabia, Lewis & Büttiker [64])	2/7 (8)	190	360	57	0.59	190	–
*P. arabicus* HESUA^c^ (Saudi Arabia, Lewis & Büttiker [64])	2/7 (8)	–	–	54 (42–69)	(0.50–0.59)	–	(12–22)
“*P.* Naqben” species (Saudi Arabia, Lewis & Büttiker 1[64])	2/7	–	–	54–98	0.58	–	(15–22)
*P. naqbenius* (Saudi Arabia, Lewis & Büttiker, [65])^d^				53 (41–69)			
*P. naqbenius* holotype (PV, unpublished)	2/7	185	357	60	–	172	16
*P. naqbenius* syntype (PV, unpublished)	2/7	196	381	80	0.6	185	21

*Notes*: Measurements were collected from: ^a^four males from Ethiopia; the holotype and syntype of *P. naqbenius* were loaned from The National History Museum, London, UK; ^b^examination of the type-series; ^c^examination of own samples from Hesua; ^d^We speculate that the authors attributed the numbers of the coxite hairs incorrectly; All measurements are in µm

### Sand fly species identification by molecular techniques

**Fig. 3:**
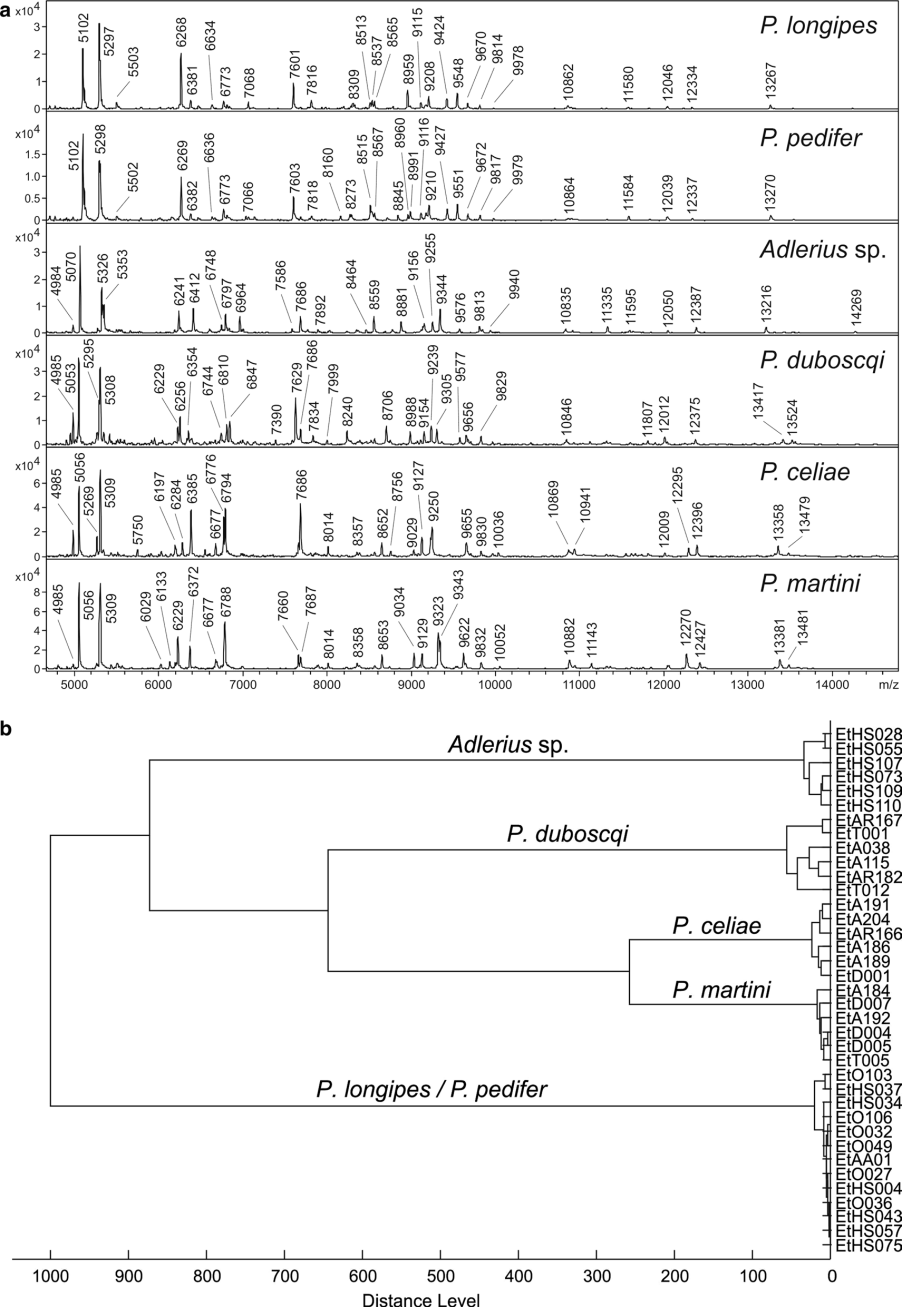
MALDI-TOF mass spectra (**a**) and the corresponding dendrogram of the protein profiles (**b**) of Ethiopian sand fly species. Zoomed mass range of 4 to 15 kDa is shown in **a** and distances in **b** are displayed in relative units

Most specimens tested gave reproducible MALDI-TOF MS spectra with a high number of intense signals (Fig. 3a). Except for the protein profiles of *P. pedifer* and *P. longipes*, each species generated a heterogeneous spectrum with species-specific peaks allowing unambiguous species identification. The dendrogram of the specimen’s protein profiles (Fig. 3b) indicates that all subgenera cluster on distinct branches. The closely related *P. martini* and *P. celiae* were clearly distinguished from each other, whereas the specimens of the subgenus *Larroussius* from all three sites grouped in a single cluster. The *Adlerius* sp. formed its own separate branch.

**Fig. 4:**
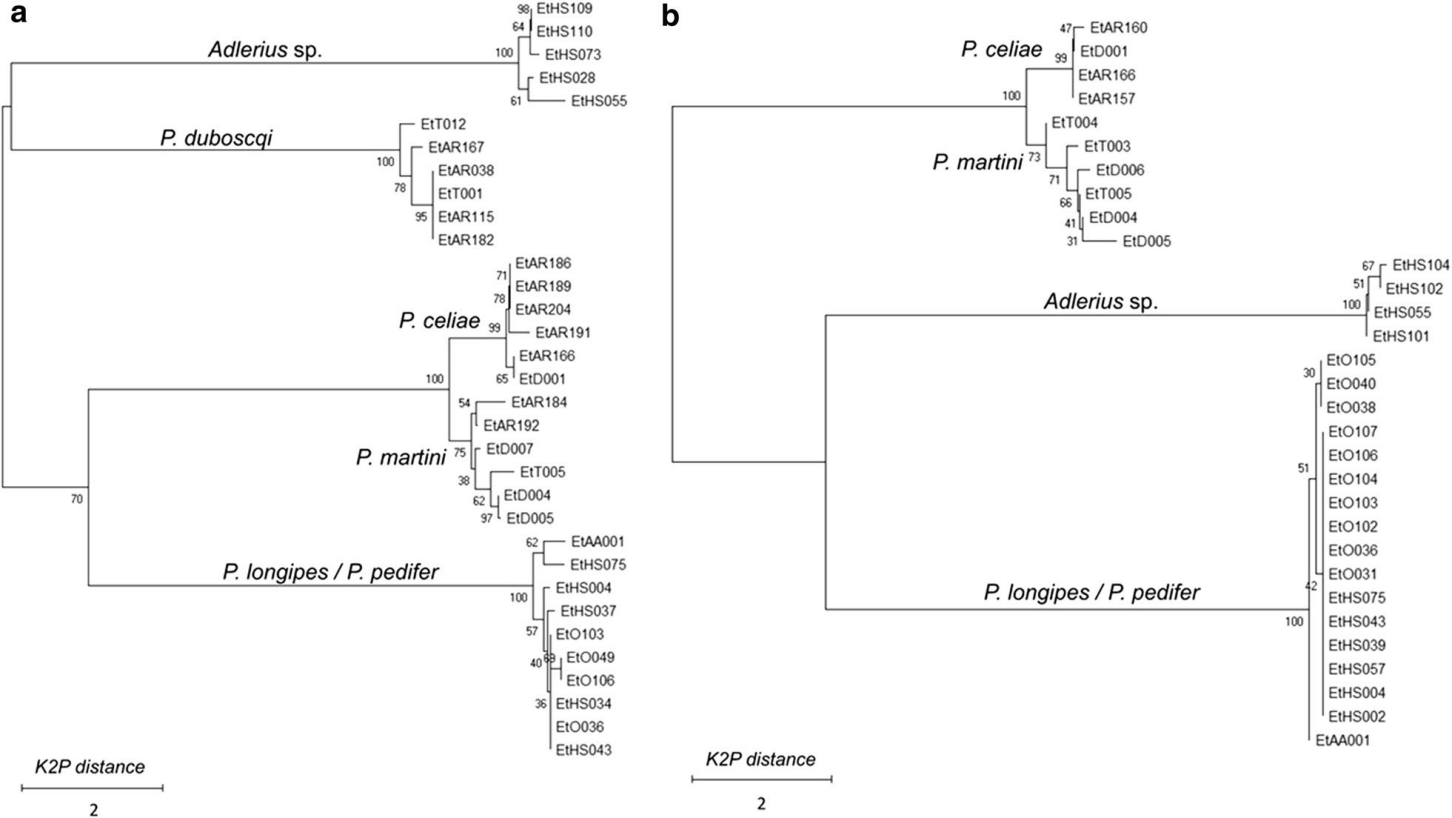
Neighbor-joining tree based on K2P distances of *cox*1 (**a**) and *nad*4 (**b**) sequences of Ethiopian sand flies. *Abbreviations:* K2P, Kimura 2-parameter model

Taxonomic clustering of the *cox*1 and *nad*4 sequences in the NJ tree (Fig. 4a, b, respectively) substantiates the hierarchical clustering of the protein profiles (Fig. 3b), separating all species except for the two *Larroussius* species. The mean intra-group K2P distances within the *P. longipes* and *P. pedifer* cluster was only 0.3% (SD 0.1) for *cox*1 and 0.1% (SD 0.1) for *nad*4 genes (Additional file 1: Table S1), indicating that there is no genetic difference between the two presumable species. The K2P distance between *P. martini* and *P. celiae* was only 1.6% (SD 0.4) for *cox*1 and 1.2% (SD 0.3) for *nad*4 genes, yet the species clearly clustered in distinct clades (Additional file 2: Table S2).

**Fig. 5:**
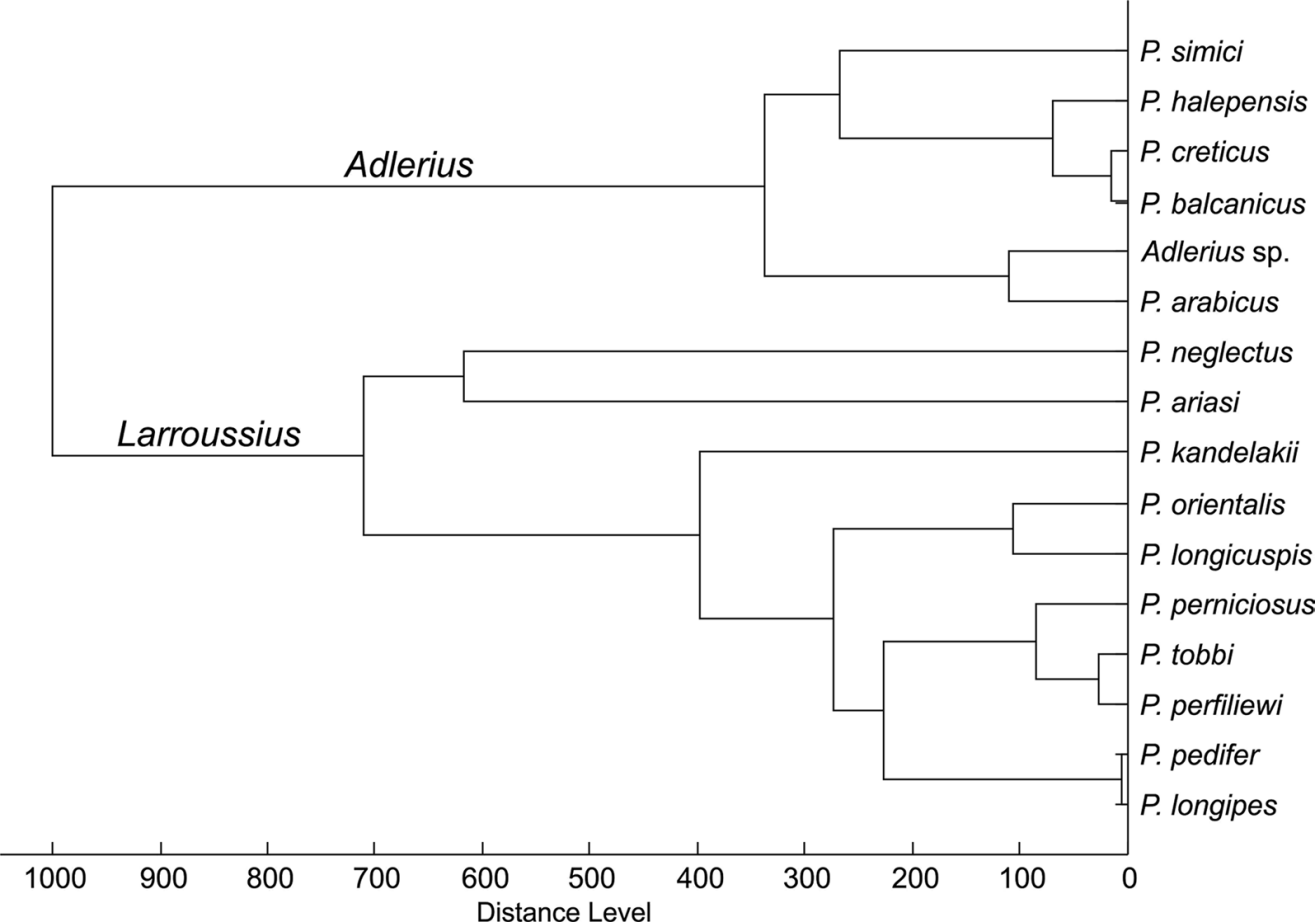
Dendrogram of MSP reference spectra of *Larroussius* and *Adlerius* sand flies from Ethiopia and our library. The origin of the species is given in the methods section. Specifically, *P. arabicus* was derived from the sand fly facility at Charles University, Czech Republic, which originated from northern Israel. Distances are displayed in relative units. *Abbreviations:* MSP, main spectrum

The dendrogram including the MSP reference spectra of *P. pedifer*, *P. longipes*, *Adlerius* sp. and other available species of the subgenera *Larroussius* and *Adlerius* from our library (Fig. 5) demonstrates that only the two Ethiopian CL vectors cannot be distinguished from each other, while the protein profiling could clearly differentiate all other analyzed species, including the closely related *P. tobbi* and *P. perfiliewi* that provide highly similar, yet species-specific protein spectra. The dendrogram depicts that *Adlerius* sp. is closely related with *P. arabicus* from Israel but some distinct peaks were found in their protein profiles (Additional file 3: Figure S1). Moreover, there is a large relative distance between the MSP reference spectra of the two species (Fig. 5), demonstrating that *Adlerius* sp. is definitely not *P. arabicus*.

**Fig. 6:**
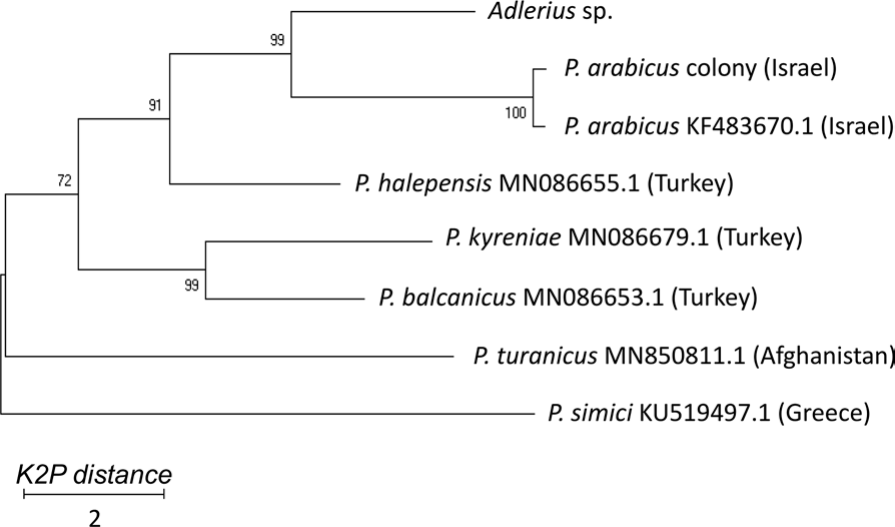
Neighbor-joining tree of the *cox*1 gene of *Adlerius* sp. from Ethiopia and other *Adlerius* species. Accession codes of the species retrieved from GenBank are displayed after the species name. The *P. arabicus* colony was obtained from Israel. *Abbreviations*: K2P, Kimura 2-parameter model

Furthermore, the *cox*1 sequence profile of the *Adlerius* sp. was compared with available GenBank sequences of other species of the *Adlerius* subgenus (Fig. 6), which supports the MSP dendrogram (Fig. 5), indicating that the species is closely related to *P. arabicus* but constitutes a distinct branch with a K2P distance of 6.5% (SD 1.2, Additional file 4: Table S3).

## Discussion

Since East Africa accounts worldwide among the regions most affected by both cutaneous and visceral leishmaniasis, phlebotomine sand flies occurring in the Horn of Africa have been extensively studied due to their exclusive role as vectors in the transmission cycles. Unfortunately, whereas considerable amount of knowledge regarding the diverse sand fly fauna of Ethiopia has been gradually gathered, the last comprehensive morphological key for species identification has not been updated for more than five decades [42], leaving the attempt to identify the specimens collected in field surveys very tedious and challenging. This further urges the need to deploy alternative molecular techniques that have recently emerged and were successfully adopted for species identification of various medically significant arthropods. In this study, we applied an integrative taxonomic approach to identify most CL and VL vectors in Ethiopia using a combination of morphological assessment, sequencing analysis of two genetic markers and MALDI-TOF MS protein profiling, and demonstrate some novelties in the complex sand fly fauna of Ethiopia.

The three vectors of *L. donovani* in Ethiopia are *P. orientalis*, *P. martini* and *P. celiae*. The former is a species of the subgenus *Larroussius*, whereas the latter two belong to the subgenus *Synphlebotomus* and occur sympatrically in Aba Roba focus in southwestern Ethiopia [32, 35]. While to our knowledge this is the only place where *P. celiae* has been identified in Ethiopia, *P. martini* is more widespread, serving as a vector also in the northwestern and southeastern parts of the country, where it sometimes cohabitates with *P. orientalis* [8, 52, 53]*.*

Males of *P. martini* and *P. celiae* can be easily distinguished based on the morphology of the lateral lobes on their external genitalia, but females of these two species were previously regarded as indistinguishable until Gebre-Michael & Lane [32] differentiated them based on the labrum length and labrum-to-wing length ratio. Moreover, the spermathecal ducts vary in length, but this characteristic cannot be used for routine identifications, as it requires dissecting out the fragile ducts. However, still a few field-caught specimens in the study of Gebre-Michael & Lane [32] could not surely be identified by these characters and the measurement ranges do not match with findings from Davidson et al*.* [54] in Egypt and the original description of *P. martini* by Parrot et al*.* [55] in Dire Dawa (about 600 km from Aba Roba in eastern Ethiopia). This could be due to the geographical distance or environmental conditions but as a result these measurements can presumably not be extrapolated to a larger area without leading to misidentifications.

*Phlebotomus martini* and *P. celiae* have a different distribution, infection prevalence and abundance, and thus their contribution to disease transmission varies [35]. Therefore, proper species identification is pivotal and requires novel determination techniques. Both genetic and MALDI-TOF MS analyses in this study pointed out that DNA marker sequences and protein profiles were similar, and enable rapid and conclusive species identification, indicating that this is a suitable approach for species identification.

Sand flies were also captured from different CL endemic sites in Ethiopia, where we found two species of the subgenus *Larroussius*, identified morphologically as *P. pedifer* and *P. longipes*. The latter species was first described by Parrot et al*.* [56] in 1939 in Ethiopia, but the species complex was re-examined by Lewis et al. [41] in 1972, who compared presumed *P. longipes* specimens from Kenya and South Sudan with *P. longipes* from different places in northern, western and central Ethiopia. The species from Kenya appeared smaller than the ones from Ethiopia, although this was probably a geographical difference. Moreover, the authors describe that male specimens from Kenya had an up-turned foot-like aedeagus, whereas it was only slightly up-turned in the Ethiopian species. Therefore, the Kenyan specimens were described as a new related species, *P. pedifer*, which was later also found as the CL vector in southwestern Ethiopia (Ochollo) [41]. Killick-Kendrick et al*.* [31] later confirmed that males of *P. pedifer* and *P. longipes* can be distinguished based on this slight difference in the aedeagus shape and additionally also the number of inner surface coxite hairs, being 50–60 for *P. pedifer* and 35–50 for *P. longipes*. In contrast, females of the two species are considered indistinguishable [30, 41], except for a slight difference in the base of the spermathecal ducts, which often still results in inconclusive identifications [31].

Results of the present study show that the tips of the aedeagi of the Ethiopian CL vectors varied among all collected specimens, being partially dependent on the orientation of the specimen on the slide. Also, the number of coxite hairs overlapped significantly between *P. pedifer* and *P. longipes* and seems more geographically dependent than species-specific. Accordingly, we conclude that these morphological characters are not appropriate for species identification. Moreover, sequences of two genetic markers and protein profiles of *P. pedifer* and *P. longipes* were found identical, whereas all other species of the subgenus *Larroussius* in our database could be easily distinguished using MALDI-TOF MS. This includes also *P. orientalis* protein profiles generated by colony specimens originating from an Ethiopian VL endemic site, Melka Werer. Even though *P. orientalis* is morphologically closely related to *P. pedifer* and *P. longipes* [51], our results show that this species can easily be distinguished based on MALDI-TOF MS protein profiling. Among other analyzed *Larroussius* species, the method also clearly differentiated between *P. perfiliewi* and *P. tobbi* which are very similar morphologically and have a similar geographical distribution [57–59], demonstrating the discriminatory power of the approach. Furthermore, the ecology of *P. pedifer* and *P. longipes* is comparable, as both occur in close association with hyraxes, inhabiting caves, basalt cliffs, cracks in boulders and gorges at mid-highland altitudes [10, 11, 34]. Collectively, our results suggest that the two species incriminated in the transmission cycle of *L. aethiopica* may actually represent a single species, *P. longipes*, which was described first.

*Phlebotomus duboscqi* was found in the Aba Roba focus and could easily be identified by MALDI-TOF MS as it matched with *P. duboscqi* from Senegal in our reference database. Two other species of the subgenus *Phlebotomus* were recorded elsewhere in Ethiopia, namely *P. papatasi* and *P. bergeroti*, which are sympatric with *P. duboscqi* in some areas [60, 61]. In these locations, taxonomic tools like MALDI-TOF MS protein profiling can be very useful for entomological studies to differentiate similar species and to thoroughly investigate their potential roles in *Leishmania* transmission.

Another *Phlebotomus* species was found in Hagere Selam, which belonged to the subgenus *Adlerius* according to the morphology of the collected specimens. The only other currently reported *Adlerius* species in Ethiopia is *P. arabicus*, which has been described in the upper Awash Valley, where it was captured from rocky valleys and found infected with *Leishmania* parasites, although the species could not be identified due to contamination [62]. In Israel, *P. arabicus* is known as an efficient vector of *L. tropica* in rocky places colonized by hyraxes [63]. Our specimens were also captured in caves together with *P. longipes.* Morphometric and molecular analyses in our study, however, show that the captured species is definitely not *P. arabicus*.

In 1980, Artemiev [37] described four males and one female sand fly from Yemen and one male and one female sand fly from Ethiopia as *P. davidi*. The Ethiopian specimens were derived from the area around Langano Lake, approximately 200 km south of Addis Ababa. Our measurements of several morphological parameters, namely number of coxite hairs and the distance from the aedeagus tip to tooth, fit best with the description of *P. davidi* [37]. However, the coxite, style and aedeagus length were slightly larger than described by Artemiev [37], which can be due to small sample sizes and geographical variation. Moreover, this author described that the antennae of the male specimen from Ethiopia were missing and that the female specimen from Ethiopia had a different pharynx than the one from Yemen, requiring more specimens from both countries to determine the true taxonomy [37]. This suggests that the species from Ethiopia was potentially different from the holotype of *P. davidi* collected in Yemen.

Lewis & Büttiker [64] questioned the validity of *P. davidi* as a species based on doubts about the value of the ascoid formula that also distinguished it from *P. arabicus*. While not addressing apparently different numbers of coxite hairs and emphasizing the overlapping values of wing lengths and possible same or varying ascoid formulae, they concluded that specimens from Ethiopia previously identified as *P. davidi* are a geographical variant of *P. arabicus* and regarded *P. davidi* as its synonym. Interestingly, in the same study, the authors collected specimens of another *Adlerius* species in several localities in Saudi Arabia, provisionally named “*P.* Naqben”, with a remarkably wide range in number of coxite hairs (54–98) [64]. In a later study, Lewis & Büttiker [65] formally described it as *P. naqbenius*. However, for this new species they do not provide the morphological characters regarded as important for the subgenus *Adlerius* by Artemiev [37] (ascoid formula, length of style, coxite and aedeagus tip to tooth) and they base its description on the number of coxite hairs, which they nevertheless now find significantly lower compared to *P. arabicus*. The authors did not discuss further this abrupt change in their understanding of *P. arabicus* and *P. naqbenius* morphology and we speculate that the authors attributed the number of coxite hairs incorrectly. It should be noted that while the validity of *P. naqbenius* was later never formally challenged, there are no recent records of this species in faunistic surveys from Saudi Arabia in the last decades and no molecular reference data are available. We hypothesize that, based on the scarce morphological data, our specimens of *Adlerius* sp. do not represent an Ethiopian population of *P. naqbenius*. On the contrary, they are morphologically more similar to the Ethiopian specimens described as *P. davidi* by Artemiev [37] and together they represent a different species from specimens collected in Yemen. This suggests that investigation on its taxonomy, distribution and potential role in *Leishmania* transmission is required.

In conclusion, MALDI-TOF MS protein profiling and DNA barcoding provided similar results in this study, demonstrating that methods of molecular taxonomy provide a viable alternative to often poorly characterized and minuscule morphological characters required for traditional species identification. An integrated approach is especially useful in regions where the sand fly fauna is highly diverse. Because DNA barcoding is much more expensive, time consuming and sequences of many Ethiopian species are not available in online genetic databases, we suggest to develop a centralized MALDI-TOF MS protein profile database and use this approach for routine identification of sand fly specimens from field surveys.

## Conclusions

To the best of our knowledge, this study demonstrates for the first time that MALDI-TOF MS protein profiling is a suitable taxonomical approach for cost-effective, unambiguous species identification of Ethiopian sand flies. DNA- and protein-based molecular techniques as well as morphometric analysis suggest that the vectors of *L. aethiopica*, *P. pedifer* and *P. longipes*, may represent a single species. We also report that the *Adlerius* species we found, differs from *P. arabicus*, the only *Adlerius* species reported in Ethiopia to date. This *Adlerius* species probably represents a new species. Collectively, our results contribute to the understanding of the complex sand fly fauna in Ethiopia, a region heavily burdened with human leishmaniasis.

## Supplementary information

**Additional file 1: Table S1.** Intraspecies K2P distances of *cox*1 and *nad*4 genes of Ethiopian sand flies. *Abbreviations:* K2P, Kimura 2-parameter model; SD, standard deviation.

**Additional file 2: Table S2.** Interspecies K2P distances of *cox*1 and *nad*4 genes of Ethiopian sand flies. The lower left quadrant presents the K2P distances (SD) of the *cox*1 gene, the upper right quadrant of the *nad*4 gene. The *nad*4 gene of *P. duboscqi* specimens was not included in the analysis^a^.

**Additional file 3: Figure S1.** Comparison of MALDI-TOF MS protein profiles of Ethiopian *Adlerius* sp. with five other species of the subgenus *Adlerius*. Zoomed mass range of 4 to 15 kDa is depicted.

**Additional file 4: Table S3** Interspecies K2P distances of *cox*1 gene of *Phlebotomus* sand flies of the subgenus *Adlerius*.

## Data Availability

The datasets analyzed during the present study are available from the corresponding authors on reasonable request.

## Abbreviations

BLAST: Basic Local Alignment Search Tool
*cox*1: Cytochrome *c* oxidase subunit 1
CL: Cutaneous leishmaniasis
K2P: Kimura two-parameter model
MALDI-TOF MS: Matrix-assisted laser desorption/ionization time-of-flight mass spectrometry
MCL: Mucocutaneous leishmaniasis
MEGA: Molecular Evolutionary Genetics Analysis
MSP: Main spectrum
NJ: Neighbor joining
SD: Standard deviation
SNNPR: Southern Nations, Nationalities, and Peoples’ Region
VL: Visceral leishmaniasis
