# Supplementary material for: An integrative approach to identify sand fly vectors of leishmaniasis in Ethiopia by morphological and molecular techniques
Source: Parasit Vectors. 2020 Nov 17;13:580. doi: 10.1186/s13071-020-04450-2 (PMC7672994; doi:10.1186/s13071-020-04450-2)
Supplement: Supplementary file 1 — Additional file 1: Table S1. Intraspecies K2P distances of cox1 and nad4 genes of Ethiopian sand flies. Abbreviations: K2P, Kimura 2-parameter model; SD, standard deviation. [file 13071_2020_4450_MOESM1_ESM.docx]

| **Sand fly species** | **K2P COI (SD)** | **K2P ND4 (SD)** |
| --- | --- | --- |
| *Adlerius* sp. | 0.5 (0.2) | 0.1 (0.1) |
| *P. longipes/P. pedifer* | 0.3 (0.1) | 0.1 (0.1) |
| *P. duboscqi* | 0.3 (0.1) | - |
| *P. celiae* | 0.2 (0.1) | 0.1 (0.1) |
| *P. martini* | 0.6 (0.2) | 0.4 (0.2) |
