# Supplementary material for: An integrative approach to identify sand fly vectors of leishmaniasis in Ethiopia by morphological and molecular techniques
Source: Parasit Vectors. 2020 Nov 17;13:580. doi: 10.1186/s13071-020-04450-2 (PMC7672994; doi:10.1186/s13071-020-04450-2)
Supplement: Supplementary file 2 — Additional file 2: Table S2. Interspecies K2P distances of cox1 and nad4 genes of Ethiopian sand flies. The lower left quadrant presents the K2P distances (SD) of the cox1 gene, the upper right quadrant of the nad4 gene. The nad4 gene of P. duboscqi specimens was not included in the analysisa. [file 13071_2020_4450_MOESM2_ESM.docx]

| **Sand fly species** | ***P. Adlerius sp.*** | ***P. longipes/P. pedifer*** | ***P. duboscqui*** | ***P. celiae*** | ***P. martini*** |
| --- | --- | --- | --- | --- | --- |
| ***Adlerius* sp.** | - | 14.9 (1.6) | ^a^ | 15.6 (1.6) | 16.0 (1.7) |
| ***P. longipes/P. pedifer*** | 15.4 (1.6) | - | ^a^ | 15.1 (1.6) | 15.1 (1.6) |
| ***P. duboscqi*** | 14.9 (1.6) | 17.5 (1.7) | - | ^a^ | ^a^ |
| ***P. celiae*** | 17.1 (1.8) | 13.7 (1.5) | 14.7 (1.6) | - | 1.2 (0.3) |
| ***P. martini*** | 16.2 (1.7) | 13.8 (1.5) | 14.0 (1.5) | 1.6 (0.4) | - |
