# Supplementary figures and images for: An integrative approach to identify sand fly vectors of leishmaniasis in Ethiopia by morphological and molecular techniques
Source: Parasit Vectors. 2020 Nov 17;13:580. doi: 10.1186/s13071-020-04450-2 (PMC7672994; doi:10.1186/s13071-020-04450-2)

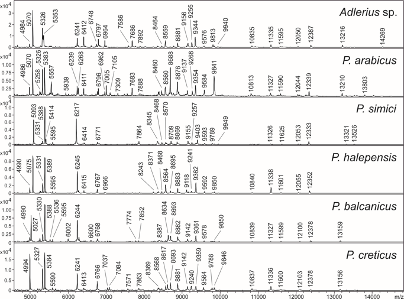

Supplement: Supplementary file 3 — Additional file 3: Figure S1. Comparison of MALDI-TOF MS protein profiles of Ethiopian Adlerius sp. with five other species of the subgenus Adlerius. Zoomed mass range of 4 to 15 kDa is depicted. [file 13071_2020_4450_MOESM3_ESM.jpg]
