# Supplementary material for: An integrative approach to identify sand fly vectors of leishmaniasis in Ethiopia by morphological and molecular techniques
Source: Parasit Vectors. 2020 Nov 17;13:580. doi: 10.1186/s13071-020-04450-2 (PMC7672994; doi:10.1186/s13071-020-04450-2)
Supplement: Supplementary file 4 — Additional file 4: Table S3 Interspecies K2P distances of cox1 gene of Phlebotomus sand flies of the subgenus Adlerius. [file 13071_2020_4450_MOESM4_ESM.docx]

| **Sand fly species** | **1** | **2** | **3** | **4** | **5** | **6** | **7** |
| --- | --- | --- | --- | --- | --- | --- | --- |
| **1. *P. turanicus***  Afghanistan, MN850811.1 |  |  |  |  |  |  |  |
| **2. *P. kyreniae***  Turkey, MN086679.1 | 13.4  (1.7) |  |  |  |  |  |  |
| **3. *P. simici***  Greece, KU519497.1 | 14.2  (1.8) | 13.1  (1.6) |  |  |  |  |  |
| **4. *P. halepensis***  Turkey, MN086655.1 | 11.1  (1.6) | 8.6  (1.3) | 12.9  (1.7) |  |  |  |  |
| **5. *P. arabicus***  Israel, KF483670.1 | 12.5  (1.6) | 12.5  (1.6) | 16.3  (1.9) | 7.5  (1.2) |  |  |  |
| **6. *P. balcanicus***  Turkey, MN086653.1 | 11.8  (1.5) | 5.7 (1.1) | 12.9 (1.7) | 7.7 (1.2) | 11.2 (1.6) |  |  |
| **7. *Adlerius* sp.**  Ethiopia, Hagere Selam | 12.5  (1.7) | 11.4  (1.6) | 15.4  (1.9) | 7.1  (1.3) | 6.5  (1.2) | 9.1  (1.4) |  |
| **8. *P. arabicus***  Israel, colony | 13.0  (1.7) | 12.5  (1.7) | 16.3  (2.0) | 7.5  (1.3) | 0.3  (0.2) | 11.2  (1.6) | 6.1  (1.1) |
